# Transporter genes identified in landraces associated with high zinc in polished rice through panicle transcriptome for biofortification

**DOI:** 10.1371/journal.pone.0192362

**Published:** 2018-02-02

**Authors:** C. N. Neeraja, Kalyani S. Kulkarni, P. Madhu Babu, D. Sanjeeva Rao, K. Surekha, V Ravindra Babu

**Affiliations:** Department of Crop Improvement, ICAR-Indian Institute of Rice Research, Rajendranagar, Telangana, Hyderabad, India; Institute of Genetics and Developmental Biology Chinese Academy of Sciences, CHINA

## Abstract

Polished rice is poor source of micronutrients, however wide genotypic variability exists for zinc uptake and remobilization and zinc content in brown and polished grains in rice. Two landraces (Chittimutyalu and Kala Jeera Joha) and one popular improved variety (BPT 5204) were grown under zinc sufficient soil and their analyses showed high zinc in straw of improved variety, but high zinc in polished rice in landraces suggesting better translocation ability of zinc into the grain in landraces. Transcriptome analyses of the panicle tissue showed 41182 novel transcripts across three samples. Out of 1011 differentially expressed exclusive transcripts by two landraces, 311 were up regulated and 534 were down regulated. Phosphate transporter-exporter (PHO), proton-coupled peptide transporters (POT) and vacuolar iron transporter (VIT) showed enhanced and significant differential expression in landraces. Out of 24 genes subjected to quantitative real time analyses for confirmation, eight genes showed significant differential expression in landraces. Through mapping, six rice microsatellite markers spanning the genomic regions of six differentially expressed genes were validated for their association with zinc in brown and polished rice using recombinant inbred lines (RIL) of BPT 5204/Chittimutyalu. Thus, this study reports repertoire of genes associated with high zinc in polished rice and a proof concept for deployment of transcriptome information for validation in mapping population and its use in marker assisted selection for biofortification of rice with zinc.

## Introduction

Rice (*Oryza sativa* L.) is the staple food crop of 50% of the world and a major energy source especially in the developing countries. Polished rice, the most preferred form for consumption, is a poor source of micronutrients especially iron and zinc [1–3]. The excess dependence on polished rice in the Asian countries was reported to be responsible for malnutrition whose daily caloric intake is mainly confined to rice [4–6].

Polished grains of most of the rice varieties have 12–14 ppm of zinc and provide only one fifth of daily recommended zinc requirement of ~15 mg (though varies across sex and age) [7–9]. Zinc constitutes an important structural, enzymatic, and regulatory component in the human metabolism and its deficiency has been ranked at fifth position among the risk factors responsible for poor health. Dietary deficiency of zinc is a substantial global public health and nutritional problem with one third of the world population at risk due to low dietary intake of zinc [10–13]. International Zinc Association reports that 49% of world’s population is affected by zinc deficiency with maximum adverse effect on children. Zinc deficiency is most notably accountable for growth retardation, stunting, impeded intellectual development, and vulnerability to diarrhoea and pneumonia (www.zinc.org/health/). In order to meet at least 25% of the estimated average zinc requirement for overcoming the most severe zinc deficiency, a breeding target of 28 ppm was set in polished rice based the nutrient needs, daily food intake, retention and bioavailability analyses (www.harvestplus.org). Thus, enhancing the zinc content of polished rice has lot of potential to address wide spread zinc deficiency problem responsible for malnutrition in developing countries. Different strategies, such as biofortification, foliar or soil application of zinc fertilizers have been suggested and demonstrated to increase the zinc content of cereals [14, 15]. Of these, biofortification, an approach of the development of micronutrient-dense staple food crops to alleviate micronutrient malnutrition is targeted, sustainable and cost effective, hence most preferred [16]. Since 2000, several attempts are being made in rice for zinc biofortification through conventional breeding and genetic engineering approaches. To develop high zinc rice lines using conventional breeding, characterization of genetic variation in grain zinc concentration is a prerequisite. Germplasm evaluation has shown genotypic differences with several folds of zinc concentration in brown and polished rice in landraces [17–21].

Using the donors for high zinc content, several breeding lines with high zinc are being developed and evaluated under HarvestPlus, international and national programs (www.harvestplus.org; www.irri.org) [22–24]. As a proof of concept of combining high zinc with high yield, varieties with high zinc in polished rice developed through conventional breeding have been nationally released in Bangladesh (2013) through HarvestPlus and in India (2016) through All India Coordinated Rice Improvement Project (AICRIP) (www.harvestplus.org; www.irri.org; www.drricar.org).

Rice grain zinc concentration is influenced by a large number of plant and environmental factors depending on external zinc supply and soil conditions [25, 26]. Several plant factors affect the uptake of zinc from soil to roots, transport to leaves and stem and remobilization to developing grains [26, 27], thus understanding their genetics and molecular mechanism would help in identifying the critical candidate genes and their deployment in the development of high zinc rice varieties [28]. Several genes/gene families involved in zinc homeostasis *viz*., zinc related transporter (*ZRT*) and iron related transporter (*IRT*) like proteins comprising *ZRT*/*IRT* protein family (*ZIP*), heavy metal ATPases (*HMA*), Yellow stripe like (*YSL*), natural resistance associated macrophage protein (*NRAMP*), metal tolerant protein (*MTP*), nicotianamine synthase (*NAS*), nicotianamine aminotransferase (*NAAT*), zinc-induced facilitator-like (*ZIFL*) and others have been characterized in rice. Several transcription factors (TF) like *OsNAC*, *OsIDEF* and *OsIRO* also showed to play an important role in up regulating the genes involved in metal homeostasis [29]. Through expression analyses, several genes were shown to be associated with enhanced iron and zinc concentrations in different tissues in rice [30–34], however, the role of different families of genes in zinc metabolism of rice yet to be elucidated [35]. It appears that the expression of genes associated with zinc metabolism varies under different situations of zinc availability either deficient or sufficient or excess in soil [36–42]. Zinc localized in the seed aleurone layer chelates with phytic acid, main form of inorganic phosphate (Pi) storage in seeds to form phytate, a salt of inositol phosphate and inhibits zinc solubility, digestibility and absorption in human body [43–45].

Though several genes associated with zinc metabolism have been characterized in rice, very little is known about how zinc is transported from leaf xylem to phloem of developing seeds and ultimately unloaded into seeds [3]. Physiological and transcriptome analyses of response to zinc deficiency of two rice lines with contrasting tolerance was reported to be determined by root growth, maintenance and organic acid exudation rates, and not by zinc-transporter activity [46], but microarray analysis of zinc deficient rice showed up regulation of several genes involved in zinc transport [39].

With the advent of time, RNA-Seq has become the most important approach to study gene expression profiling using the next generation sequencing technologies providing a more precise measurement of gene transcripts dynamics on global scale in different tissues and biological contexts [47]. Rice landraces are known source of many desirable traits and have been characterized with high zinc in polished rice, although low yielding as opposed to improved varieties [19, 20]. In general, to find out the genes associated with traits of interest in rice landraces, the transcriptome studies comprise differential treatments of susceptible and tolerant genotypes (mostly landraces) under stressed conditions *viz*., drought, salt and cold, deficient or excess nutrients versus control conditions for the differential expression of genes [48–53]. With an objective of identification of the genes exclusively associated with high zinc in polished rice, those would be efficient in regular rice growing conditions as prevail in the farmers’ fields, the present study was conducted under regular or zinc sufficient situation. To identify the genes responsible for higher zinc in polished rice, RNA-Seq based transcriptomic analyses of developing panicle of two landraces and a widely grown popular variety with differential zinc in the polished rice were compared and a set of 311 up regulated genes and 534 down regulated genes in two landraces were identified and the six promising genes were validated through quantitative real time PCR (qRT-PCR) and their association with zinc in polished rice through mapping.

## Materials and methods

### Plant material and their growth conditions

Based on their differential zinc content in polished rice, three genotypes *viz*., BPT 5204 (BPT), Chittimutyalu (CTM) and Kala Jeera Joha (KJJ) were selected for RNA-Seq. BPT 5204 also known as Samba Mahsuri, is a medium slender grain *indica* rice variety, very popular with farmers and consumers across India because of its high yield and excellent cooking quality [54]. Chittimutyalu and Kala Jeera Joha are land races, though low yielders, and still are cultivated in some parts of India for their high aroma and quality. Moreover, Chittimutyalu is a national check for zinc content (>20 ppm) in polished rice and BPT 5204 is a national check for yield (~15 ppm) under All India Coordinated Rice Improvement Program (AICRIP) Biofortification trials [22]. Kala Jeera Joha has also shown high level of zinc in polished rice (>20 ppm) across locations in our studies. The details of agro-morphological, yield and quality related parameters of the three genotypes are given in S1 Table (S1 Fig). BPT 5204, Chittimutyalu and Kala Jeera Joha were grown in pots at polyhouse and the pots were arranged in a complete randomized block design with three replicates. The developing panicles just before booting were excised in three replications for each genotype and stored in RNAlater (Invitrogen, USA) at -80°C.

Using BPT 5204 and Chittimutyalu as parents, recombinant inbred lines (RILs) were developed using single seed descent method. A set of 300 RILs (F_8_) were grown in two replications in an augmented block design at the research farm of ICAR-Indian Institute of Rice Research (IIRR), Hyderabad during wet season 2011 following recommended package of practices. The experimental soil characteristics were: pH 8.2; non-saline (EC 0.7l dS/m); calcareous (free CaCO_3_ 5.01%); CEC 44.1 C mol (p+)/kg soil and medium soil organic carbon (0.69%); low soil available nitrogen (228 kg ha^-1^); high available phosphorus (105 kg P_2_O_5_ ha^-1^), high available potassium (530 kg K_2_O ha^-1^), and high available zinc (12.5 ppm) [55].

### Estimation of zinc content

#### Grain samples

The seed of all the panicles from three plants and the mapping population (from the middle row in case of field experiments) was harvested, pooled and divided into three parts to be analyzed as three replicates. The seeds were dehusked using JLGJ4.5 testing rice husker (Jingjian Huayuan International Trade Co., Ltd) sponsored by HarvestPlus and polisher (Krishi International India Ltd.) with non ferrous and non zinc components. Each sample of brown and polished rice (5 g) was subjected to energy dispersive X-ray fluorescent spectrophotometer (ED-XRF) (OXFORD Instruments X-Supreme 8000) at ICAR-IIRR as per standardized protocols [56].

#### Straw samples

The straw samples of three genotypes (in replication) were washed, dried at 60° C and powdered using non-ferrous and non-zinc grinder. Triacid mixture (10 mL) with nitric, sulphuric and perchloric acid (9:4:1) (Merck) was added into 1 g of the powdered sample in the digestion tube and the sample was heat digested for two hours. The resulting residue was filtered and made up to 50 mL for measuring the iron and zinc content using atomic absorption spectrophotometer (Varian Model AA240).

### RNA isolation and Illumina NextSeq 500 sequencing

The total RNA was isolated by Trizol (Invitrogen, USA) method from the developing panicles in three replications and pooled for RNA-seq. The yield and purity of RNA were assessed by measuring the absorbance at 260 and 280 nm and the quality was checked using RNA 6000 Nano Assay Kit on Agilent Bioanalyzer 2100 for RNA Integrity Number (RIN) values. The samples having RIN above 8.5 were only processed further to ensure quality of the RNA-seq data. RNA sequencing of the six samples was performed by Sandor Lifesciences Pvt. Ltd., Hyderabad. The pair end sequencing libraries were prepared using Illumina NextSeq 500 RNA Library Preparation Kit as per manufacturer's protocol (Illumina®, San Diego, CA). Total RNA used to isolated from each sample poly(A) mRNA followed by first strand cDNA synthesis second strand cDNA synthesis, adaptor ligation, 75-bp cDNA fragments isolation and amplification. Library quality control and quantification were performed.

### Pre-processing and reference mapping

The raw reads were filtered to obtain high-quality reads by trimming adapters and low quality bases (> Q20). Rice genome and gene information for reference cultivar Nipponbare (*Oryza sativa* L. subsp. *japonica*) was downloaded from Ensemble (http://plants.ensembl.org/Oryza_sativa/Info/Index). The resulting high-quality reads were mapped onto the downloaded reference genome. Sequencing reads from all three samples were mapped to the rice genome sequence scaffolds using TopHat (V 2.0.13) (mate inner distance and mate standard deviation: 600; Segment length: 42). The resulting alignment (in BAM file format) was used to generate transcript annotations (GTF format) with the Cufflinks (V 2.2.1). These read counts were used in statistical tests of differential expression between test and reference for differential gene expression. The sequence reads were submitted to GenBank database under accession number SRX26113645 (http://www.ncbi.nih.gov).

### Differential expression of transcripts

Differences in gene expression between the six samples were tested with Cuffdiff (v 2.2.1) package of Cufflinks using FPKM (Fragments Per Kilobase of transcript per Million mapped reads) from reference-guided mapping. Transcripts with 1.5 fold change (up and down regulated), false discovery rate (FDR) of 0.005 and p-value ≤0.05 were considered as significantly expressed. The differential expression of transcripts belonging to particular functional classes was represented as heat map using Multi experiment Viewer (MeV) v4.9.0 using hierarchical clustering with Pearson Uncentered correlation and complete linkage method [57].

### Functional annotation of transcripts and metabolic pathway enrichment

GO analysis and GO enrichment was performed using Ensemble Rice (*Oryza sativa*) GO database using perl script. KAAS (KEGG Automatic Annotation Server) was used to functionally annotate differentially expressed genes by BLAST comparisons against KEGG GENES database [58].

### Mapping differentially expressed transcripts with quantitative trait loci (QTL)

The differentially expressed transcripts among the genotypes were mapped to the regions of metaQTLs (mQTLs) reported for iron and zinc content in rice for knowing their co-localization with QTL [59].

### Validation by qRT-PCR

The differentially expressed transcripts with functional annotation and high fold values were selected for validation through qRT-PCR. The fasta sequences of transcripts were retrieved and input in batch primer3 online tool by selecting the generic option and following criterion: product size of 100–200 (bp), primer size of 18–22 nts, melting temperature 59–62°C, rest of the parameters were default and the primer sequences of all genes used in this study are listed in S2 Table [60, 61]. All the primers used in the study were synthesized at Integrated DNA Technologies (USA). The stability of endogenous reference genes at panicle initiation stage was analyzed using RefFinder (http://www.leonxie.com/referencegene.php) as per the program guidelines. For qRT-PCR, the total RNA was isolated from the panicle tissues just before booting for each genotype in two biological replications grown in pots under polyhouse. The first strand cDNA was synthesized from using RevertAid First Strand cDNA Synthesis Kit (Thermoscientific) and served as template for qRT-PCR. Each reaction (10 μl SYBR Green, 3 μl template cDNA, 0.4 μl each of the primers (10 μM), and 6.2 μl RNase-free water) was performed in triplicate with the following program 95°C (2 min) followed by 40 cycles of 95°C (5 s), 60°C (30 s) with fluorescent signal recording and 72°C for 30 s in 7500 Applied Biosystems® 7500 Real-Time PCR System. Melting curve analysis was performed to check the primer specificity. The data were analyzed using 7500 Sequence Detection Software (Applied Biosystems, USA). Expressed protein (LOC_Os06g43650.1) (forward primer: GGTAGACATCAGTGCCAGGAA and reverse primer: CTGAGAGGTTCCAACACAAGC) was used as endogenous gene for relative expression of transcripts for validation [62]. The gene expression was calculated using the 2-ΔΔCt method and the expression of BPT 5204 with low zinc in polished rice was taken as control and Chittimutyalu and Kala Jeera Joha were considered as treated [63].

### Mapping of differentially expressed genes to high zinc in polished rice with rice microsatellite (RM) markers

For six differentially expressed candidate genes, 9–12 RM markers were selected from each of the genomic region spanning ~145–344 kb for chromosomes 1, 3, 4, 7, 8 and 11 of the candidate gene (www.gramene.org) (S2 Table). Parental polymorphism with 61 RM markers was studied between BPT 5204 and Chittimutyalu and the polymorphic markers were surveyed in 300 RILs of BPT 5204 and Chittimutyalu. DNA isolation, PCR amplification of the RM markers, gel electrophoresis and documentation were carried out as per earlier protocols [64]. The marker-trait associations in RIL were identified using ANOVA 1 command of MapDisto v 1.7 [65].

### Sequencing of a candidate gene

Targeting Os07g0257200 encoding putative metal transporter *NRAMP5* based its differential expression, 21 candidate gene based primers were designed encompassing the complete gene (S2 Table). The polymorphic products derived from BPT, CTM and KJJ along with two set of six selected RIL with low zinc and high zinc were also subjected to sequence analysis. PCR products were eluted from 1% agarose gel, purified using Wizard® SV Gel and PCR Clean-Up System (Promega); cloned in pGEM-T easy vector (Promega) and sequenced using an ABI Prism 3700 automated DNA sequencer (Perkin Elmer, MA) by Integrated DNA Technologies (USA). The derived consensus sequences were compared between three genotypes and also with the sequences of 12 RILs along with reference genome using CLUSTALW multiple sequence alignment tool employing MEGA 7 software (www.megasoftware.net). The sequence difference as indels (insertions/deletions) and Single Nucleotide Polymorphisms (SNPs) between the parents were considered for polymorphism (S3 Table). The overall work done is represented in S2 Fig.

## Results

Transcriptomics analyses of developing panicles of three genotypes comprising two landraces with high zinc content (>20 ppm) and one improved variety with zinc content (~12 ppm) in polished rice grown under normal conditions revealed a set of differential expressed genes comprising several families associated with mineral homeostasis and other activities of cell metabolism.

### Zinc content in rice grain in three genotypes and mapping population

The two landraces showed high zinc in brown and polished rice in comparison to BPT, whereas high zinc content was observed in straw of BPT 5204 than landraces (Table 1). In the RIL mapping population of BPT/CTM, the zinc content ranged from 15.5 to 48.3 ppm in brown rice and 8.4 to 43.1 ppm in polished rice.

**Table 1:** Zinc content (ppm) (mean ± SD) in straw and grain samples of BPT, CTM and KJJ.

	Straw		Brown rice		Polished rice	
	Replication 1	Replication 2	Replication 1	Replication 2	Replication 1	Replication 2
**Zinc**						
BPT 5204	50.65±9.28	46.28±5.16	14.15±0.78	13.78±0.26	8.7±2.12	8.2±0.33
Chittimutyalu	30.90±4.53	38.61±10.14	30.8±1.41	28.87±1.37	25.25±4.03	22.68±1.81
Kala Jeera Joha	41.59±9.62	37.72±1.90	31.7±5.3	32.03±3.83	28.7±3.68	28.9±3.39

### RNA-Seq/sequencing statistics

The RNA-Seq of BPT, CTM and KJJ at panicle initiation stage emanated 116298544 total raw reads. The raw reads were further processed for 106296448 high quality reads by removing the low quality reads. The alignment results of high quality reads on rice genome showed 83.6%, 82% and 86% alignment respectively (Table 2). Out of 41182 transcripts expressed across three genotypes, 1860 transcripts exclusively present in BPT, 1436 transcripts were exclusively present in CTM and 945 genes in KJJ.

**Table 2:** Summary statistics of reads.

Genotypes	Total number of raw reads	High quality filtered reads	Low quality reads	% high quality reads	Reads Aligned	% Reads Alignment
BPT 5204	42923678	39374194	3549484	91.73	32908327	83.6
Chittimutiyalu	38111630	34545258	3566372	90.64	28328289	82.0
Kala Jeera Joha	35263236	32376996	2886240	91.81	27830601	86.0

### Differential expression of transcripts

**Fig 1:**
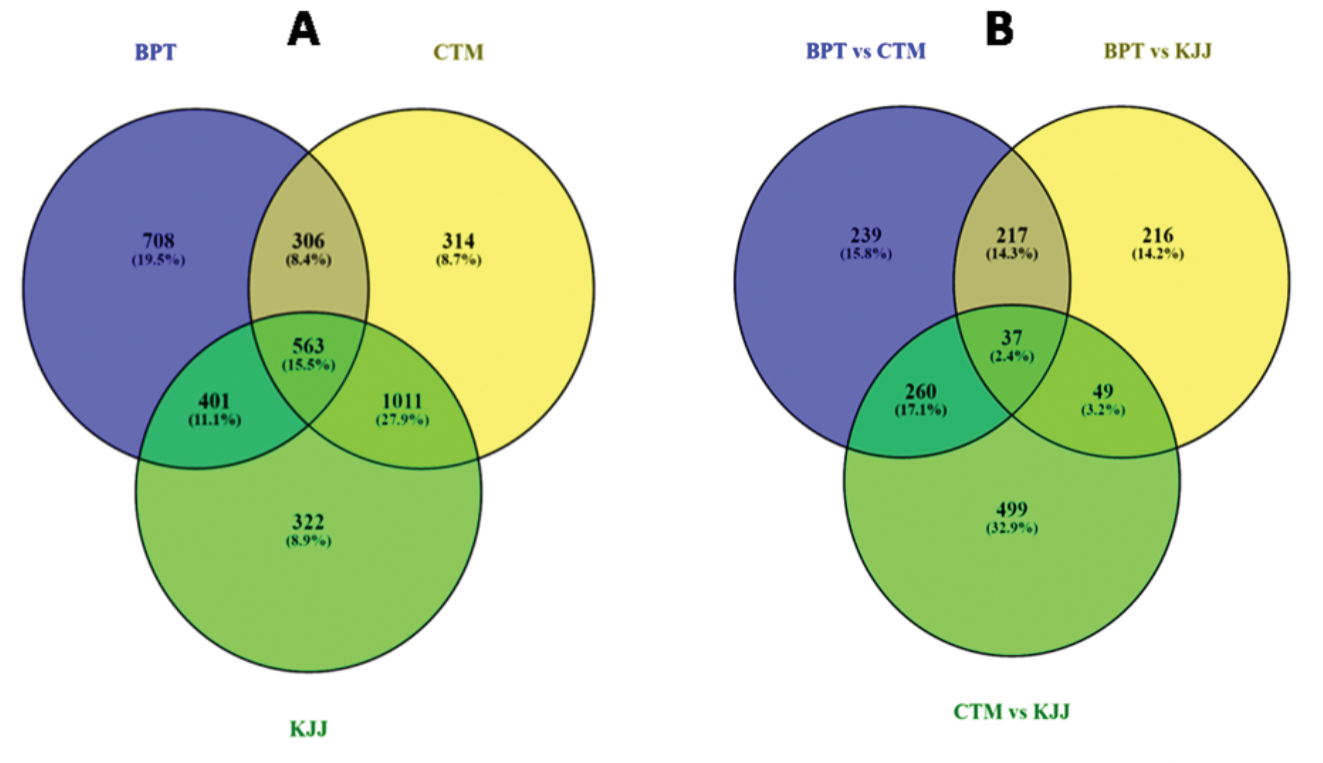
Number of differentially expressed transcripts. Number of transcripts in each genotype (A) and total number of differentially expressed transcripts in combination (B).

**Fig 2:**
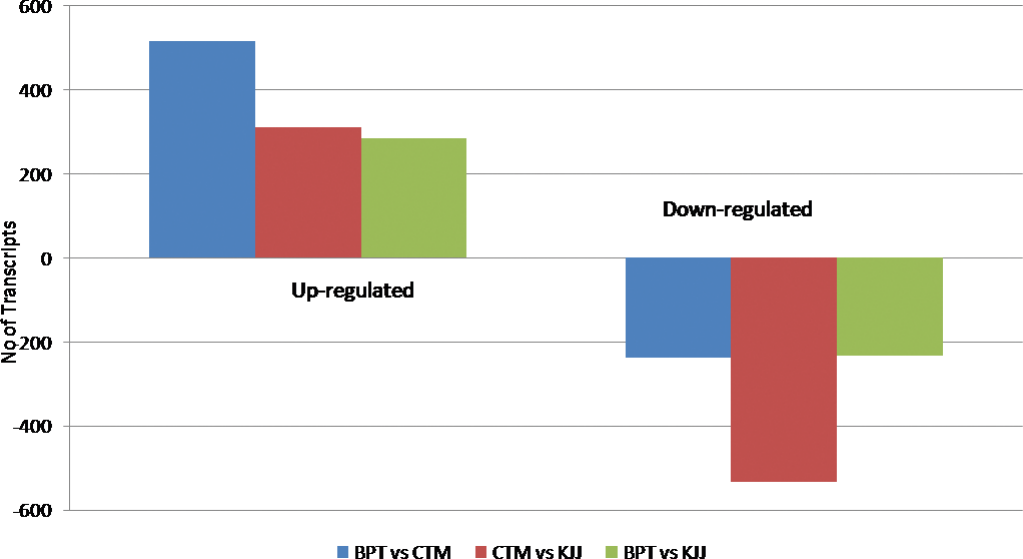
Number of significantly up and down regulated transcripts. Pair wise comparisons of up and down regulated transcripts in BPT, CTM and KJJ.

Differential expression of transcripts showed 563 common transcripts in BPT, CTM and KJJ with 708 transcripts exclusive in BPT, 314 transcripts exclusive in CTM and 322 exclusive in KJJ (Fig 1A). Comparative transcriptome analysis showed a set of common transcripts of 401 between BPT and KJJ, 306 between BPT and CTM and 1011 between BPT and KJJ. Among the three, there were only 37 common transcripts with 217 common transcripts between the comparisons of BPT/CTM and BPT/KJJ; 260 common transcripts between the comparisons of BPT/CTM and CTM/KJJ and 49 common transcripts between the comparisons of BPT/KJJ and CTM/KJJ (Fig 1B). The exclusive transcripts between the comparisons were 239 in BPT/CTM, 216 in BPT/KJJ and 499 in CTM/KJJ. The maximum number of transcripts was observed for CTM/KJJ followed by BPT/CTM and BPT/KJJ. Comparative transcriptome analysis among the rice cultivars revealed that a total of 311 up-regulated and 534 down-regulated transcripts were common in both CTM and KJJ (Fig 2).

The maximum up regulated transcripts were observed in BPT/CTM and maximum down regulated transcripts were observed in CTM/KJJ. Some of the interesting genes with higher log fold change (log_2_RPKM ratio > 10) *viz*., OS02G0810900-putative *NAC* domain protein *NAC1*; OS01G0270300-peroxidase super family protein; OS01G0276500-histidine biosynthesis bifunctional protein; OS01G0363300-CASP-like protein 1E1; OS01G0578000-DNA repair protein radA (RadA)-like; OS02G0198700-putative subtilisin-like proteinase AIR3; OS02G0620100-kinetochore protein-like; OS02G0703900-nodulin-like protein; Os05g0594700-putative early-responsive to dehydration stress protein (ERD4); OS07G0249600-Putative transcription factor; OS07G0538200-Putative serine/threonine-specific protein kinase; OS08G0543400-putative hydroxyl anthranilate hydroxycinnamoyltransferase 2; OS08G0547100-probable 6-phosphogluconolactonase 3, chloroplastic; OS09G0110300-cyclase-like protein; OS09G0419500-Putative ubiquitin conjugating enzyme 7 interacting protein; OS11G0150100-phosphoglycerate mutase family protein, expressed; OS12G0503000-ureide permease 4, putative including many other uncharacterized proteins. Three of the transcripts *viz*., Os03g0271100 protein-plastid sigma factor 2B; OS11G0704300-probable GTP-binding protein OBGM, mitochondrial OS12G0113500CBL-interacting protein kinase 14 showed up as well down regulation across the genotypes.

### Transporters

**Fig 3:**
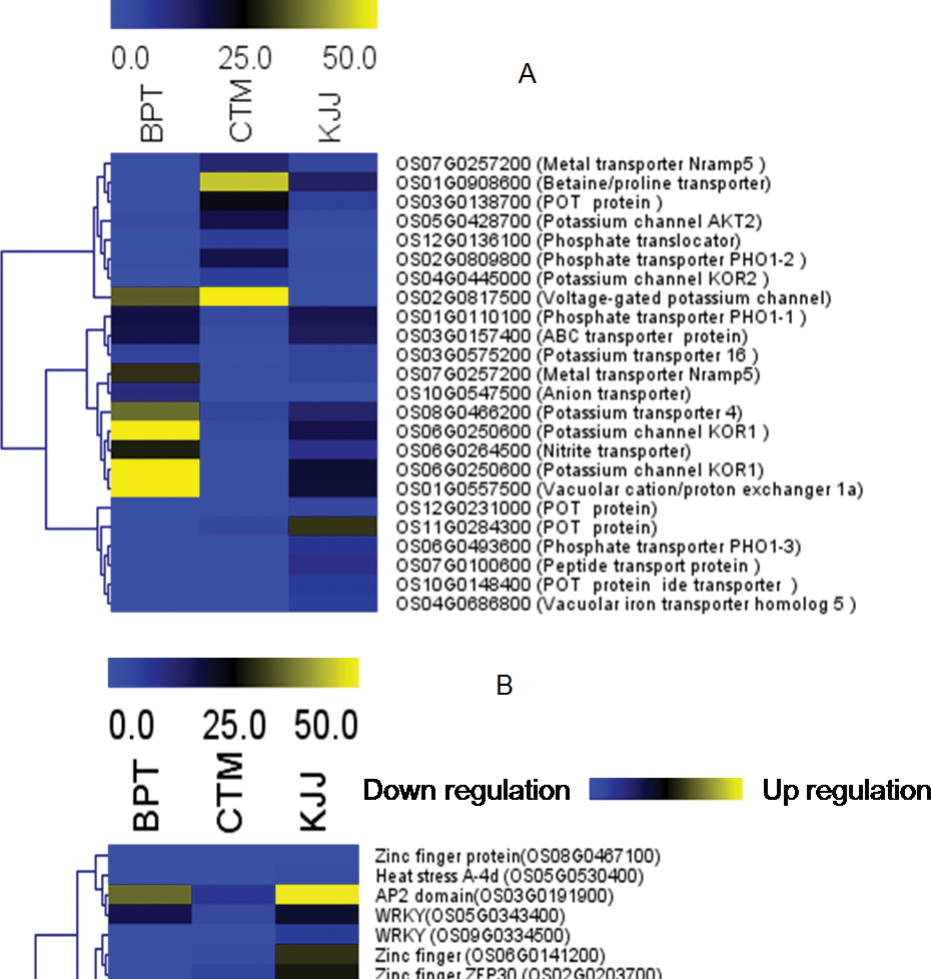
Representation of differentially expressed transcripts. Differentially expressed transporters in BPT 5204, CTM and KJJ (A) Differentially expressed transcription factors in BPT 5204, CTM and KJJ (B).

Out of the transcripts exclusively expressed in two landraces (CTM and KJJ) with high zinc content in polished rice, four *POT* family proteins showed differential expression along with a putative peptide transport protein. Two earlier reported genes associated with iron and zinc metabolism *viz*., *NRAMP5* and vacuolar iron transporter (*VIT*) also showed up regulation only in landraces. Out of two genes with enhanced expression from another interesting gene family of phosphate exporters, *PHO 1–3* showed enhanced expression only in landraces, whereas *PHO1-1* expressed in all three genotypes. Several other transporter gene families showed enhanced expression in BPT, an improved variety (Table 3) (Fig 3A).

**Table 3:** Significant differentially expressed transcripts of transporter gene families.

	Gene_description	BPT	CTM	KJJ
OS01G0908600	Betaine/proline transporter	** -**	44.54	14.53
OS07G0257200	Metal transporter *NRAMP5*	** -**	12.59	4.49
OS06G0493600	Phosphate transporter *PHO1-3*	** -**	0.60	8.14
OS11G0284300	*POT* family protein	** -**	4.30	30.18
OS12G0231000	*POT* family protein	** -**	1.06	4.57
OS10G0148400	*POT* family protein expressed transporter	** -**	0.88	7.07
OS03G0138700	*POT* family protein, expressed	** -**	23.06	6.15
OS07G0100600	Putative peptide transport protein	** -**	0.29	10.03
OS04G0686800	Vacuolar iron transporter homolog 5	** -**	0.85	6.55
OS02G0809800	Phosphate transporter *PHO1-2*	1.56	16.99	2.42
OS05G0428700	Potassium channel *AKT2*	2.79	17.42	3.02
OS03G0157400	*ABC* transporter family protein	17.14	2.17	15.59
OS01G0110100	Phosphate transporter *PHO1-1*	17.82	3.70	16.67
OS08G0466200	Potassium transporter 4	35.76	4.92	13.55
OS06G0250600	Potassium channel *KOR1*	57.65	2.86	17.39
OS03G0575200	Potassium transporter 16	5.44	0.46	5.02
OS06G0250600	Potassium channel *KOR1*	66.27		19.67
OS06G0264500	Putative nitrite transporter	27.83		9.4
OS01G0557500	Vacuolar cation/proton exchanger 1a	72.77		19.2
OS03G0411800	Zinc transporter 2	28.12		2.79
OS12G0136100	Phosphate translocator	0.23	6.32	
OS04G0445000	Potassium channel *KOR2*	0.49	6.66	
OS02G0817500	Voltage-gated potassium channel subunit beta	33.93	33.57	
OS10G0547500	Putative anion transporter	11.62	0.38	

### Transcription factors (TFs)

Out of the transcription factor families with different expression in landraces, two *WRKY* family transcription factor genes, one zinc finger gene showed increased expression along with one *ZIM* motif family protein in landraces (Fig 3B). In our study, one transcript for *NAS3 viz*., OS07G0689600-nicotianamine synthase 3 was present in both the landraces with three fold up regulation in KJJ as compared to CTM. Some of the uncharacterized transcripts *viz*., EPlOSAT00000010656, EPlOSAT00000031121, EPlOSAT00000050720, EPlOSAT00000050731, EPlOSAT00000050742 and EPlOSAT00000050753 also showed exclusive and differential expression in landraces.

### Pathway enrichment

**Fig 4:**
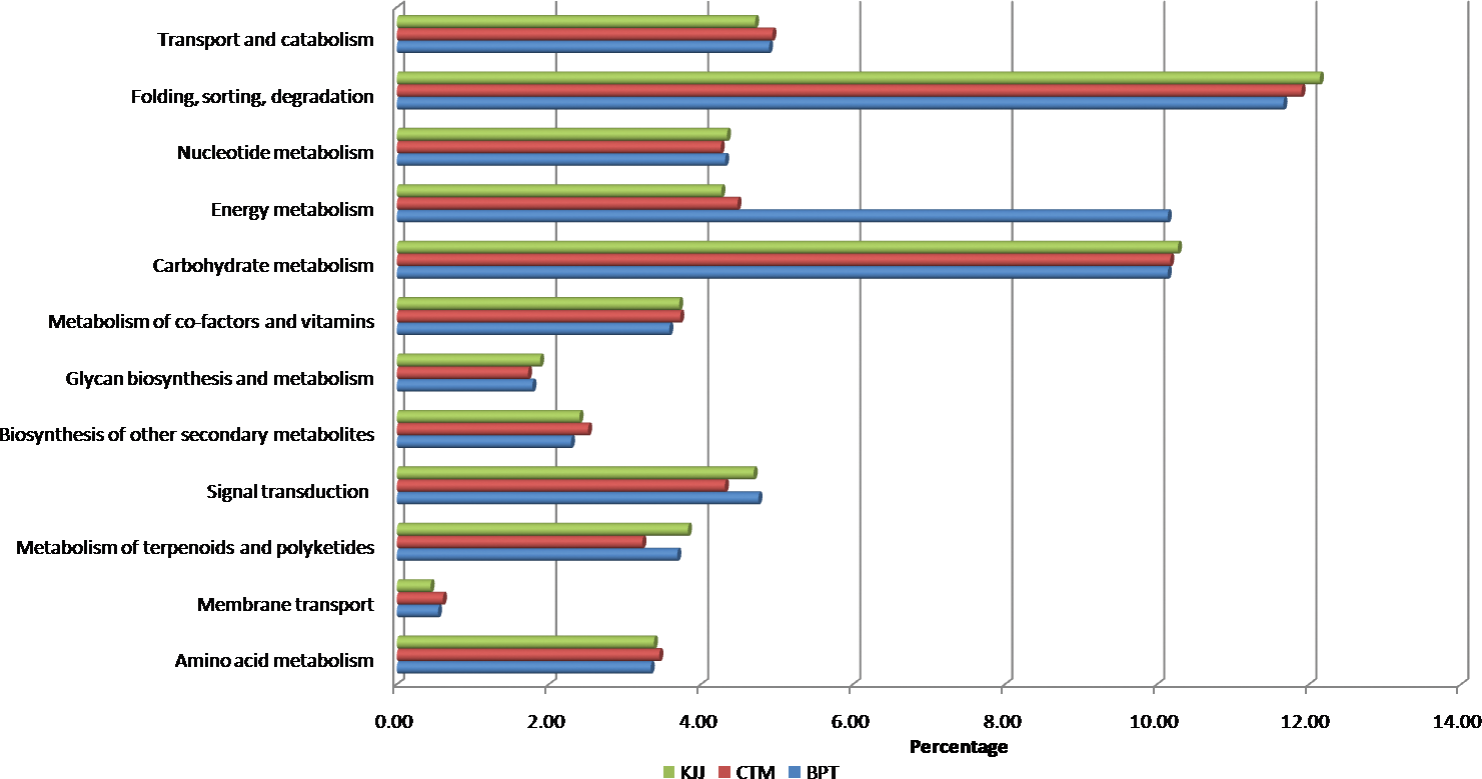
Pathway enrichment. Differentially expressed transcripts for pathway enrichment using KAAS annotation server.

The GO enrichment analysis was performed for differentially expressed transcripts among the three genotypes to gain more insights into their involvement in various biological processes for accumulation of high zinc in polished rice. Transcripts related to amino acid metabolism, membrane transport, metabolism of terpenoids and polyketides, signal transduction, biosynthesis of other secondary metabolites, glycan biosynthesis and metabolism, metabolism of co-factors and vitamins, carbohydrate metabolism, energy metabolism, nucleotide metabolism, folding, sorting, degradation and transport and catabolism were highly expressed. Higher percentage of transcripts in CTM and KJJ than BPT were mapped to amino acid metabolism, biosynthesis of other secondary metabolites, carbohydrate metabolism, folding, sorting and degradation pathways (Fig 4) (S3 Fig).

### Validation through qRT-PCR

**Fig 5:**
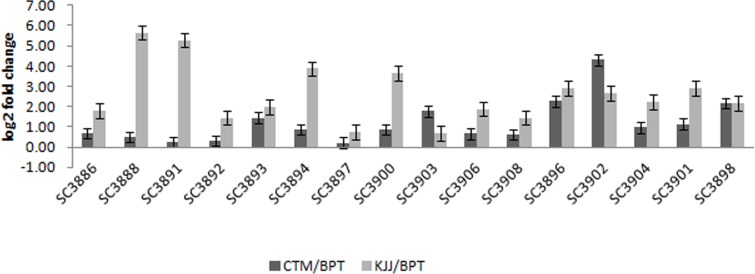
Relative expression of transcripts through Real-time PCR. Up regulation/High expression (SC3886:Sodium/calcium exchanger protein; SC3888:Homeobox associated leucine zipper;SC3891:ZOS3-18 C2H2 Zinc finger protein; SC3892:MYB family transcription factor; SC3893:MATE efflux family protein; SC3894:Vauolar iron transporter homolog5; SC3897:Potassium channel SKOR; SC3900:Peptide transporter; SC3903:OsFBX261; SC3906:WRKY74; SC3908:metallothionein, SC3896: Putative integral membrane protein; SC3902:NAS Nicotianamine synthase; SC3904:SHR5_receptor; SC3901:Metal transporter NRAMP5; SC3898:Putative nitrite transporter. Bars on top show the standard error of three biological replications. Expression normalized with endogenous gene.

The differential expression values of all the selected transcripts obtained by qRT-PCR analysis were plotted along with the RNA-Seq data. Validation of 24 up and down regulated transcripts involved in zinc homeostasis like transporters, transcription factors, signalling and secondary metabolite pathways through qRT-PCR showed significant differential expression for eight genes. Differential expression of transcripts of three genes *viz*., Os03g0839200-*MATE* efflux family protein, Os04g0686800-vacuolar iron transporter homolog 5 and Os07g0257200-metal transporter *NRAMP5* was observed in landraces (Fig 5). Increased expression of Os01g0783800-serine/threonine-protein kinase, Os07g0100600-putative peptide transport protein, Os11g0570000- receptor kinase in CTM and Os02g0649300-homeobox-leucine zipper protein *HOX24* and Os03g0764100- zinc finger transcription factor *ZF1* in KJJ was observed.

Based on 22 mQTL reported by Jin et al (2015), 24 differentially expressed genes were mapped to the reported QTL on chromosomes 1, 2, 3, 5, 7, 8, 10 and 12. Seven transcripts of both landraces; 12 transcripts of BPT and CTM and five transcripts of BPT and KJJ mapped to QTL associated with zinc, iron and phytate. We found differentially expressed transcripts for *MYB*, *bHLH*, serine threonine kinases and RING zinc finger proteins co-located with the reported QTL (S4 Table).

### Association of differentially expressed genes to high zinc in polished rice with rice microsatellite (RM) markers

Out of 61 RM markers identified for genomic regions spanning six differentially expressed candidate genes, ~50% polymorphism was observed between BPT 5204 and Chittimutyalu. Based on their resolution on agarose gel electrophoresis, six RM markers were screened in 300 RILs and the single marker analyses showed significant associations with zinc content of brown and polished rice validating the genomic regions with the differentially expressed transcripts (Table 4).

**Table 4:** RM markers from the genomic regions of differentially expressed genes and their association with zinc in brown and polished rice of RIL population.

			Zinc	
Associated RM marker	Gene ID	Gene Description	Brown	Polished
RM11743	Os01g0783800	Serine/threonine-protein kinase	4.13*	3.14
RM16183	Os03g0839200	*MATE* efflux family protein	0.01	0.00
RM17673	Os04g0686800	Vacuolar iron transporter homolog 5	0.59	0.67
RM21333	Os07g0257200	Metal transporter *NRAMP5*	8.61**	8.42**
RM22578	Os08g0203700	Putative Receptor-like serine/threonine kinase(*RFK1*)	0.05	0.32
RM26976	Os11g0570000	Receptor kinase	8.69**	8.16**

*significant P<0.05

**significant P<0.01

### Sequencing of *NRAMP 5* gene

Out of 21 primer pairs designed, four primers showed polymorphism, however their resolution into the parental alleles in RILs was poor on agarose gel. Hence, one polymorphic gene product of parents along with each set of six RILs with low and high zinc contents was sequenced. Upon sequence comparison of 649 bp PCR product of BPT and CTM of *NRAMP5* alleles with reference sequence Nipponbare showed several SNPs and 4 bp indel between BPT and CTM (S3 Table).

## Discussion

Biofortification of rice for high zinc in rice appears to be promising strategy for addressing the some of the malnutrition issues in developing countries, especially for those whose major diet is polished rice with poor micronutrients. The development of varieties with high zinc would be relevant to alleviate malnutrition, but the lack of information on translocation of nutrients from vegetative tissues to grains is one of the barriers to rice biofortification [40, 66, 67]. Several donors for high zinc in polished rice have been identified through the evaluation of landraces and are being used in development of high zinc breeding lines [18–21, 68]. In parallel, several studies are being conducted on mechanism of zinc uptake and its translocation into the grain [41, 42, 69–73]. However, the information on genes associated with zinc uptake and its translocation are very limited in rice, but for reports on zinc transporters and ZIP genes [27, 74–76]. Some transgenics of rice with metal chelating molecules like nicotianamine, IRT, 2’-deoxymugineic acid (DMA) targeted for the enhanced iron content also showed increased zinc content in grain, thus role of a few candidate genes in zinc homeostasis is available [27, 77–83]. Since, physiological studies of zinc in rice have shown transfer of zinc from the vegetative tissues to reproductive tissues to be constraint for achieving high zinc in rice grain, we attempted to characterize a set of genes expressed in developing panicles of two landraces (CTM and KJJ) with high zinc in the polished rice in comparison with a popular improved high yielding rice variety with low zinc content in polished rice (BPT) grown under sufficient zinc soil conditions. Most of the work on zinc nutrition in plants has concentrated on genes and pathways related to extreme phenotypes, such as zinc deficiency and zinc excess-derived changes in growth and/or bulk concentrations in shoots or roots [35, 39, 84]. In general, to identify the differential expressed genes associated with nutrients, excess or deficient conditions are studied along with control [46, 48, 50]. However, to identify the set of genes responsible for high zinc under general irrigated rice cultivation conditions as practiced by the farmers with fertilization of zinc or native soil zinc, the genotypes were grown under regular soil with sufficient zinc. The two rice landraces of the present study appeared to be promising donors for the high zinc content in polished rice reiterating the fact that the landraces to be the source of novel genes/alleles for traits of interest as observed for stress tolerance and other traits in rice [85,86].

Out of the three genotypes, zinc in straw content was ~18 to 30% more in BPT than CTM and KJJ, however, the zinc content in grain was ~ 42 to 45% more in brown rice and 30 to 35% in polished rice of CTM and KJJ suggesting the possibility of efficient translocation of zinc into grains by landraces than BPT. Similar trend of differential translocation of zinc has been reported by Johnson-Beebout et al. in two genotypes *viz*., in IR68144, a substantial amount of zinc stored in the stem has not translocated into grain, whereas in another genotype, IR69428 has more zinc in the grain even with lower concentration of zinc in the stem[73]. Wide genotypic variation of zinc uptake, its content in stems, leaves, panicles and grains has been reported in rice [41, 71]. Wissuwa et al. concluded that grain zinc concentration is largely determined by genotype rather than by zinc fertilization, which could be attributed to differences in zinc uptake behavior [26]. The mechanism of translocation of zinc into grains also appears to be different based on the zinc availability (sufficiency versus deficiency) [36, 37, 40, 69, 73]. The studies on zinc concentrations of panicle and stem between flowering and grain maturity stages suggested a relative transfer barrier between the vegetative and reproductive tissues, but for the same set of genotypes the zinc concentrations of grain were higher than the concentrations of the panicle implying that the loading of the grain from the panicle to grain is easier than loading of the panicle from the stem and sheath [41]. Thus, in the present study we could identify two landraces with efficient mechanism of translocation of zinc from stem into the reproductive tissue.

An interesting phenomenon called dilution, in case of decrease of nutrient concentrations in plant tissues with the dry matter increase is generally observed in cereals [87], thus explaining the inverse relationship of zinc content and yield [88]. In the present study, in the mapping population of BPT and CTM, we could identify ~10 promising lines with desirable recombinants of high yield and zinc content (>28 ppm).

To identify the genes associated with differential zinc content of polished rice in the panicles among the three genotypes, the whole transcriptome of three rice cultivars was analyzed and a large number of differentially expressed genes along with novel transcripts associated with trait of interest were identified. The RNA-Seq of BPT, CTM and KJJ of developing panicle before booting stage resulted in 106296448 high quality reads with 82–86% of alignment attributed to mapping of *indica* genome using *japonica* reference genome (Table 2). Mapping of reads through the transcriptome studies of *indica* or *japonica* or wild species of rice to the reference genome Nipponbare ranged from 69% to 98% based on the subspecies, stage and tissue of the genotypes [51, 52, 89]. The overall transcripts are more in BPT, an improved variety; however the exclusive transcripts are more in the landraces, thus proving that the native germplasm to be the resource of novel alleles/genes for several traits of interest in rice. The pair wise comparison of genotypes for common and exclusive transcripts also confirmed the abundance of exclusive transcripts in landraces.

More number of transcripts was up regulated in BPT as compared to both landraces and the number of the down regulated genes appears to be more in the landraces supporting the similar observations in landraces N22 and Pokkali under control conditions [89]. The conscious selection for more yield and other favourable agro-morphological traits during the development of improved varieties could have played role in the pooling of many up regulated genes for their expression in terms of phenotype. Only a small fraction of transcripts was found to be differentially regulated in this study from the analyses of the data confirming the earlier reports that at a particular stage for particular tissue, only a fewer number of stage and tissue specific differential transcripts are observed in rice [90].

Differential expression of transcripts was observed for all the three genotypes of the study. Among the three, there were only 37 common transcripts for pair wise comparisons with 563 common transcripts for genotype wise comparisons. Most of the differentially expressed genes in landraces are uncharacterized proteins, suggesting the existence of novel genes/alleles in the landraces.

Interestingly, not many obvious candidate genes associated with zinc metabolism found to be up or down regulated in the present study. Similar observations were also reported by Astudillo-Reyes et al. in their transcriptome study of developing pod of two common bean genotypes with contrasting zinc concentration grown under regular (zinc sufficient) conditions [91]. The information about *OsZIP* genes during different stages of flowering and seed development reported to be scarce but for *ZIP* genes in anthers. *Insilico* analyses has shown enhanced temporal expression of *OsNAS1*, *OsNAS3*, *OsNAAT1*, 2’-deoxymugineic acid synthase 1 (*OsDMAS*) during the flowering and seed development of Nipponbare, reference genotype of rice [27]. However, in our transcriptome study of three genotypes, differential expression of these genes was not observed in the panicle tissue at that time point of sample collection. Little genotypic variation in transcript abundance of zinc responsive root zinc transporters, P-type ATPases, *HMA*, *OsYSL*, *MTP1* and *MTP3* was observed between the RIL46 (a zinc deficiency tolerant line) and IR74 (a zinc deficiency sensitive line) [46]. However, microarray analysis of zinc deficient rice with root and shoot tissues revealed the up regulation of several genes involved in zinc transport [39]. The threshold levels of detection of differential expression needs to be compared between RNA-Seq and microarray for the zinc metabolism genes. Studies showed differential expression of candidate genes associated with zinc metabolism in flag leaves in genotypes with differential zinc, but expression studies of the association of candidate genes of zinc metabolism in panicle are few in rice [31, 92].

Two known genes with their association with zinc and iron metabolism *viz*., *NRAMP5* and Vacuolar Iron Transporter (*VIT*) also showed up regulation only in CTM and KJJ, which were further validated by qRT-PCR analyses. The *NRAMP* family of transporters appears to regulate nutrient export from the vacuole [93]. The role of rice *NRAMP5* has been characterized in manganese, iron and cadmium transport in root tissues [94, 95] and differential expression of *NRAMP5* in root tissues of genotypes with differential iron and zinc was also reported [32]. In the context of high zinc content in rice grain, *NRAMP5* may play a critical role in zinc homeostasis/mobilization of zinc from panicle to grain. Transcript for *VIT* homolog 5 involved in showed significant up regulation in landraces. Vacuolar sequestration is another mechanism to enhance the concentrations of iron and zinc in seeds [96]. Transporters belonging to several different families transport metals between the cytoplasm and the vacuole including the vacuolar membrane transporters *viz*., *OsVIT1* and *OsVIT2* to modulate Zn^2+^ and Fe^2+^ import to the vacuole and translocation between flag leaves and seeds in rice. Disruption of the rice *VIT* orthologues (*OsVIT1* and *OsVIT2*) increases iron and zinc accumulation in rice seeds and decreases iron and zinc in the source organ flag leaves, probably because VIT genes are highly expressed in rice flag leaves [97–99]. Thus, it can be hypothesized that the activity of *VIT5* could contribute to high zinc in polished rice.

The interesting observation of four proton-coupled peptide transporters (*POT*) family proteins with differential expression exclusively in landraces suggests for their role of possible nutrient metabolism. The POT/PTR family proteins are mainly involved in cellular uptake of small peptides and route the uptake of amino acids and nitrogen. The up regulation of peptide transporters corroborated well with the higher fold expression in CTM and KJJ as compared to BPT through qRT-PCR. In the phloem, zinc is thought to be transported either as Zn–NA or complexed with small proteins [42, 100]. Proteins that transport micronutrient–NA complexes have been identified recently as YSL proteins, which are members of the oligopeptide transport (OPT) family [100–102]. A proton-coupled symporter ZMYS1, was shown to function for the uptake of phytosiderophore and nicotianamine–chelated metals in maize [103]. The role of POT family in the nutrient metabolism though reported, further characterization and cloning of these genes is needed for confirming their exact mechanism of action [104]. Higher expression of the PHO exporters *viz*., *PHO 1–3* in landraces may suggest their possible function for the micronutrient concentration in the panicle tissue. The involvement of OSPHO1; 1 in the regulation of iron transport through integration of phosphate and zinc deficiency signaling in rice has been already reported [105]. Out of three rice *PHO1* genes identified, only *OsPHO1;2* was shown to play a key role in the transfer of Pi from roots to shoots and regulated by Pi deficiency, while *OsPHO1;1*, and *OsPHO1;3*, are still to be characterized [106]. Among other transporter genes potentially linked to altered zinc nutrition, nine putative phosphate transporters showed increased root expression in zinc deficiency tolerant rice line under zinc deficiency [46]. The role of *PHO* transporters in zinc metabolism as observed in this study is to be elucidated in detail, *PHO* genes could play role in tripartite nutrient PiZnFe interaction in plants [105, 107].

Out of the five transcripts for potassium channel and transporters, *AKT2* was up regulated in CTM and KJJ than BPT, but the *KOR 1* and *KOR 2* transcripts functioning as voltage-gated potassium channel were up regulated in BPT than the landraces. Since, zinc is an essential micronutrient for plant; its uptake is needed for general plant metabolism as evident from the higher concentrations of zinc in the vegetative parts in BPT, an improved variety (Table 1). However, our interest is of the genes associated with high zinc in polished rice playing role in the translocation of zinc to the reproductive tissue. Though the potassium transporters are characterized, their role in micronutrient uptake and mobilization is yet to be explored [108]. In our study, the higher expression suggests its potential for characterization for involvement in micronutrient or zinc content of plant metabolism.

Transport proteins embedded within membranes are key targets for improving the efficiency with which plants take up and use water and nutrients [109]. Various transcripts for *ABC*, *NRAM*P, phosphate, potassium, peptide, vacuolar iron transporters showed differential expression in our data and we suggest their possible role for the high zinc content in polished rice (Table 3). The transporters which are comparatively up regulated can be assumed to be actively involved in metabolic processes at panicle initiation stage. The uptake of mineral elements is mediated by various transporters belonging to different transporter families. Thus, the plant transporters can be effectively deployed for improving the uptake of nutrients and water as to enhance the yield and micronutrient in the grains [74].

Differential expression of Os03g0839200-*MATE* (multidrug and toxic compound extrusion) efflux family protein was also observed in both landraces. MATE effluxer genes were overrepresented among genes that were differentially expressed in roots between two rice genotypes with differential response to zinc deficiency under different zinc conditions and were hypothesized to be possible candidates for organic acid (OA)/DMA efflux transporters [46]. Natural variation at the *FRD3 MATE* transporter locus revealed cross-talk between iron homeostasis and zinc tolerance in *Arabidopsis* [110]. Several other genes *viz*., kinases, peptide transport protein, homeobox-leucine zipper protein, transcription factor were also up regulated in landraces and are under being validation.

Among the transcription factors, OS02G0810900 Putative NAC domain protein NAC1 showed significant log fold change and confirmed by qRT-PCR analyses in the present study. Differential transcripts for zinc finger (*ZF*) (five), *WRKY* (four), *MYB* (two), *AP2*, *bHLH*, *EREB*, *ZIM* and heat responsive TFs were also found. The transcripts for *ZF TF*, *ZFP30*, *WRKY 5* and *ZIM* motif TF showed exclusive expression only in the landraces. Transcription factors are integral in linking sensory pathways to many responses. Core sets of transcription factor family genes are differentially expressed in earlier studies including basic leucine zipper (*bZIP*), *WRKY*, *MYB*, basic helix-loop-helix (*bHLH*), and NAC families have been reported in nutrient homeostasis studies in rice. These transcription factors, in turn, regulate the expression levels of various genes that may ultimately influence the nutrient content in rice [111]. Nishiyama et al. reported that metal-chelate complexes are formed in rice phloem sap and this transport is critical for grain zinc content. The observed enhanced expression of one *NAS3* transcript (OS07G0689600) in the present study could be playing an important role in the accumulation of zinc in rice grain [112]. One of the important gene families associated with nutrient remobilization from source organs to developing seeds is the *NAC* (*NAM*, *ATAF*, and *CUC*) family of TFs [113]. Enhanced expression of *OsNAC5* expression in flag leaves and panicles and its association with higher seed iron and zinc concentrations was reported earlier in rice [31, 114]. Ricachenevsky et al. discussed in detail about the role of *NAC* factors in relation to leaf senescence with iron and content in the seed [115]. The validation of the *NAC* 1 gene for its association in zinc content in polished seeds is under progress. Among the zinc finger TFs, the expression of *ZF1* was higher in KJJ than CTM and BPT predicting its role during panicle development. Though the role of zinc- finger transcription factors in the important biological processes of plants has been studied, their involvement in zinc metabolism in grains is yet to be confirmed [116, 117]. The role of *WRKY* factors is nutrient metabolism is being explored recently. The *WRKY 74* has been shown to play regulatory role in phosphate uptake and mobilization [118, 119]. On similar lines, it can be proposed that *WRKY5* and other *WRKY* factors could be involved in regulating the zinc metabolism/ translocation in rice. Two members of the basic region/leucine zipper motif (*bZIP*) transcription factor gene family, *bZIP19* and *bZIP23*, were shown to coordinate the adaptation of *Arabidopsis* to low zinc phytoavailability [120]. The *bHLH* TF has been reported to regulate OsIRO2 play an important role in iron homeostasis [121]. In this study also, the expression of bHLH is evident of regulating the genes involved in zinc homeostasis but it would need further characterization. The TF APG found to be highly expressed in KJJ is a typical bHLH transcription factor that acts as negative regulator of grain size (grain length and weight by controlling cell elongation in lemma and palea) [122]. The role of these TFs till now has been characterized to some extent in rice roots, but their involvement during panicle initiation or grain filling and their association with nutrient uptake is yet to be elucidated. The MYB TF has been reported in the interconnection between zinc and inorganic phosphate homeostasis in *Arabidopsis*, namely the *MYB* transcription factor *PHR1*, the Pi exporter *PHO1* [123]. In our study also, *PHO* transporters and its homologues are highly expressed. Hence, it can be put forward that this *MYB* TF highly expressed in KJJ can be a candidate gene for zinc mobilization from the panicle to the rice grain. The controlled and regulated uptake and mobilization of micronutrients is very essential for maintaining homeostasis and ionic concentration in the cell. High concentration of metal may lead to toxicity and disturbances in its cellular function, thus the metal-responsive transcription factors have been reported to regulate trace metal metabolism. Moreover, TFs have been shown to play a critical role in the regulation of the levels of protein, zinc and iron in the mature grain [124]. In this study, genes from eight TF families were identified to be associated with the trait of interest, whose validation is in progress.

Some of the genes with significant fold change as indicated *viz*., peroxidase, nodulin-like protein and others can also be explored for their function in the zinc metabolism. Characterization of differentially expressed uncharacterized transcripts is also being targeted as they may play an important role in accumulation of zinc in polished rice. Through GO enrichment analysis, we conclude that out of 12 pathways, the amino acid metabolism, biosynthesis of other secondary metabolites, carbohydrate metabolism, folding, sorting and degradation pathways may contribute significantly to enhanced zinc content in polished rice.

The co-localization of 24 differentially expressed transcripts for *MYB*, *bHLH*, serine threonine kinases, RING zinc finger proteins with mQTL reported for zinc, zinc and phytate corroborated with the up regulated transcripts for phosphate transporters/ translocators in the landraces [59].

In order to validate the information of the differentially expressed genes generated in this study in deployment in marker assisted selection (MAS) for high zinc in polished rice, candidate gene based markers were developed for a differentially expressed gene, *NRAMP5* as a proof of concept. Though polymorphism was observed, the resolution of candidate gene based markers into parental alleles in the RIL population of the study was poor on agarose gel and the sequence information of the polymorphic product could not generate efficient marker system. Thus, RM markers spanning six differentially expressed genes confirmed with qRT- PCR were selected for their validation for association with traits of interest. And out of six genes, five differentially expressed genes showed association with zinc content in brown and polished rice, thus validating the differential expressed genes and their association (Table 4). Thus, we have shown the deployment of transcriptome data for the generation of the differentially expressed genes from the novel germplasm sources and their utility as markers system for MAS in our study.

The reported association of zinc and iron content in the grain suggests some level of common regulatory mechanisms for their metabolism [45], so analyses of both elements was done for plant samples (including straw and grain) and mapping population (grain). However, iron content in polished rice is much below the target iron content set by Harvestplus, thus, only limited analyses was done for iron in the present study (S5 Table).

The development of zinc biofortified rice is challenging due to the complexity of genetic and metabolic networks controlling the homeostasis of zinc [38, 111, 125]. Genotypic variability needs to be characterized for the uptake, remobilization and concentration of zinc in polished rice, which are affected by use efficiency of zinc source-sink relations [40]. Several transporter gene families appear to be associated with high zinc metabolism in polished rice. Thus, in present study, we have identified two landraces with promising zinc content in polished rice and we have validated the differential expressed genes identified through transcriptomic studies in the mapping population. Further studies are needed to target earlier and later developmental time points along with different tissue samples to better characterize genotypic differences in zinc remobilization with focus on functional characterization of zinc transporters *in planta*, elucidation of zinc uptake and sensing mechanisms, and on understanding the cross-talk between zinc homeostasis and other physiological processes [35].

## Conclusion

Our study provided an overview of the panicle transcriptome of three rice genotypes with differential zinc content in polished rice and highlighted putative candidate genes associated with high zinc in polished rice. Several novel transcripts have been identified along with the significant differentially expressed specific transporters *viz*., *NRAMP*, *VIT*, *POT*, *PHO* and *MATE*. The association of six differential expressed genes with zinc in polished rice is validated through expression and mapping analyses. We have demonstrated the generated transcriptome information for validation of associated genes in mapping population as a proof of concept. Overall, the resource generated in this study can be used to identify the suitable candidate genes for association and validation for high zinc in polished rice.

## Supporting information

S1 Fig Grains of KJJ, CTM and BPT. (TIFF)

S2 Fig Flow chart of the RNA-Seq and mapping experiments for differential expressed genes and their validation. (TIF)

S3 Fig Classification of BPT 5204, CTM and KJJ transcripts on the basis of cellular components, molecular functions and biological processes. (TIF)

S1 Table Details of agro-morphological traits, yield and quality parameters of BPT 5204, Chittimutyalu and Kala Jeera Joha across years. (XLSX)

S2 Table List of primers used for qRT-PCR, RM SSR and candidate gene. (XLSX)

S3 Table Sequence assembly of PCR product (~649 bp) of two landraces (Chittimutyalu and Kala Jeera Joha) in comparison with BPT 5204 and their segregation in RIL with high and low zinc. (XLSX)

S4 Table Significantly differentially expressed transcripts located in reported QTL region. (XLSX)

S5 Table A. Iron content (ppm) (mean ± SD) in straw and grain samples of BPT, CTM and KJJ. RM markers from the genomic regions of differentially expressed genes and their association with zinc and iron in brown and polished rice of RIL population. B. RM markers from the genomic regions of differentially expressed genes and their association with iron in brown and polished rice of RIL population.
(XLSX)
