# Correction: Transporter genes identified in landraces associated with high zinc in polished rice through panicle transcriptome for biofortification

**DOI:** 10.1371/journal.pone.0196160

**Published:** 2018-04-16

**Authors:** C. N. Neeraja, Kalyani S. Kulkarni, P. Madhu Babu, D. Sanjeeva Rao, K. Surekha, V. Ravindra Babu

The bottom of Fig 3 is cut off. The authors have provided a corrected version here.

**Fig 3:**
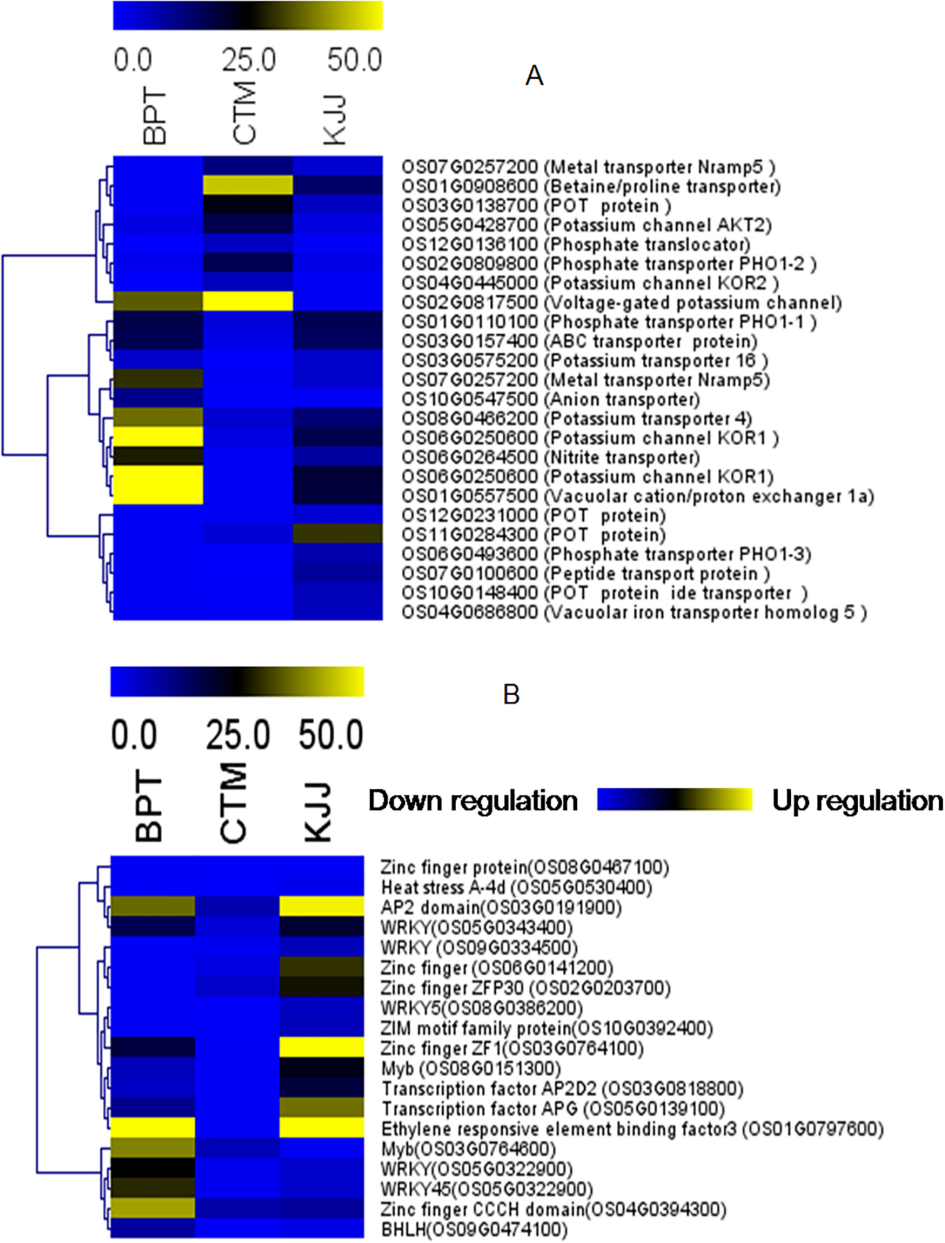
Representation of differentially expressed transcripts. Differentially expressed transporters in BPT 5204, CTM and KJJ (A) Differentially expressed transcription factors in BPT 5204, CTM and KJJ (B).
