# Supplementary figures and images for: Transporter genes identified in landraces associated with high zinc in polished rice through panicle transcriptome for biofortification
Source: PLoS One. 2018 Feb 2;13(2):e0192362. doi: 10.1371/journal.pone.0192362 (PMC5796704; doi:10.1371/journal.pone.0192362)

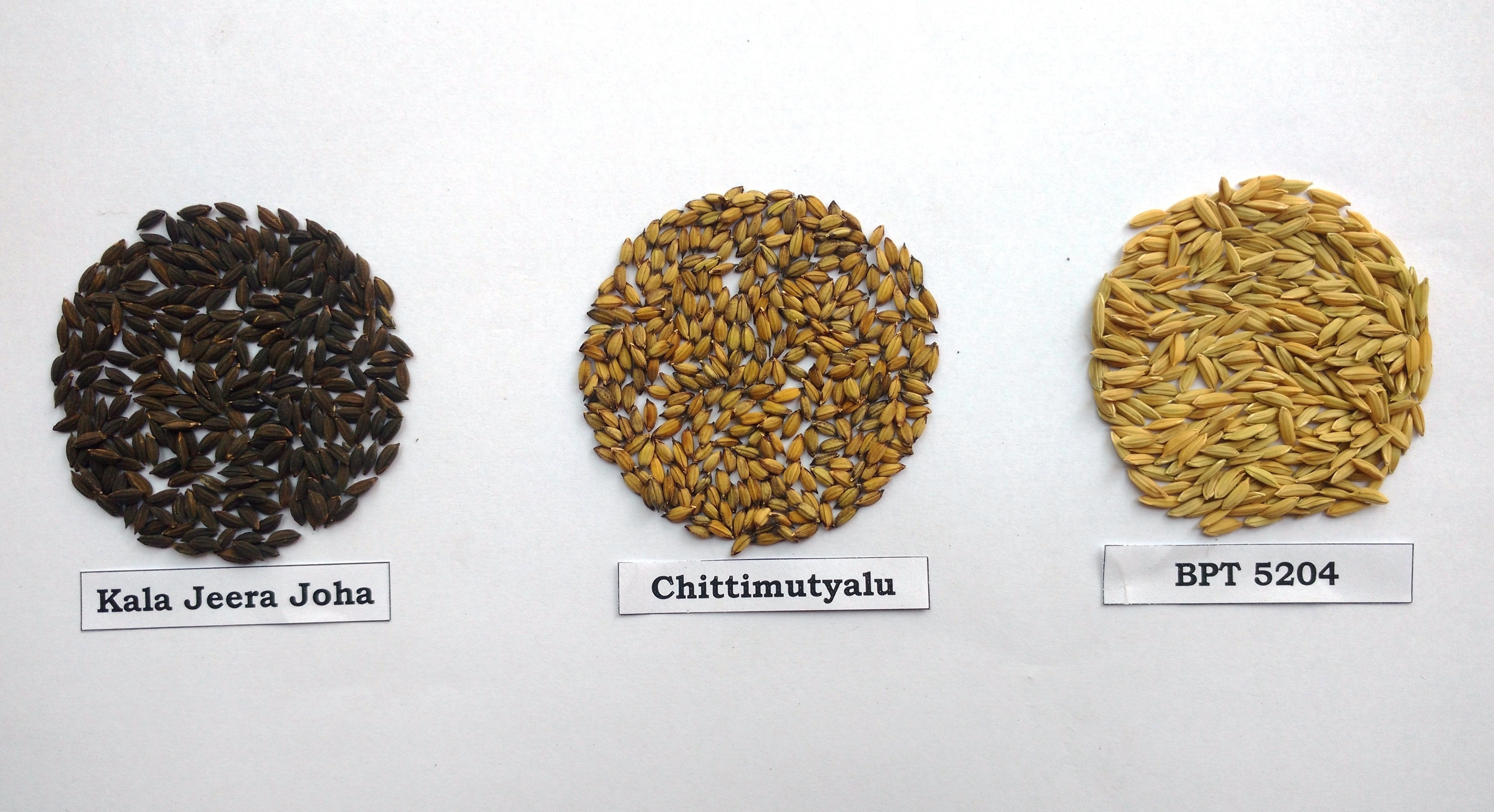

Supplement: S1 Fig — (TIFF) [file pone.0192362.s001.tiff]

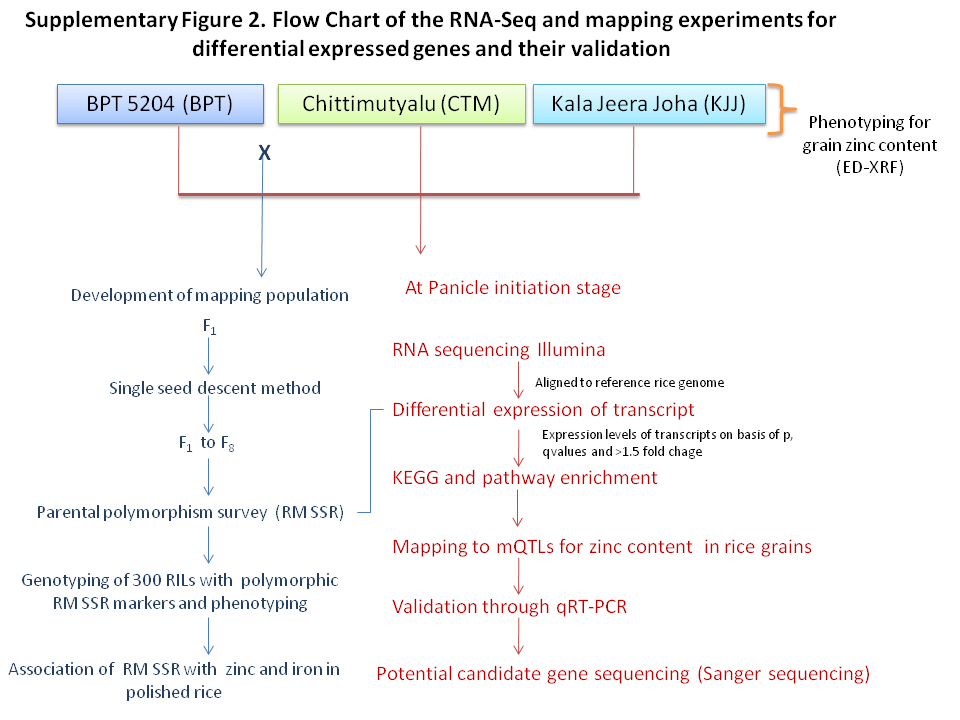

Supplement: S2 Fig — (TIF) [file pone.0192362.s002.tif]

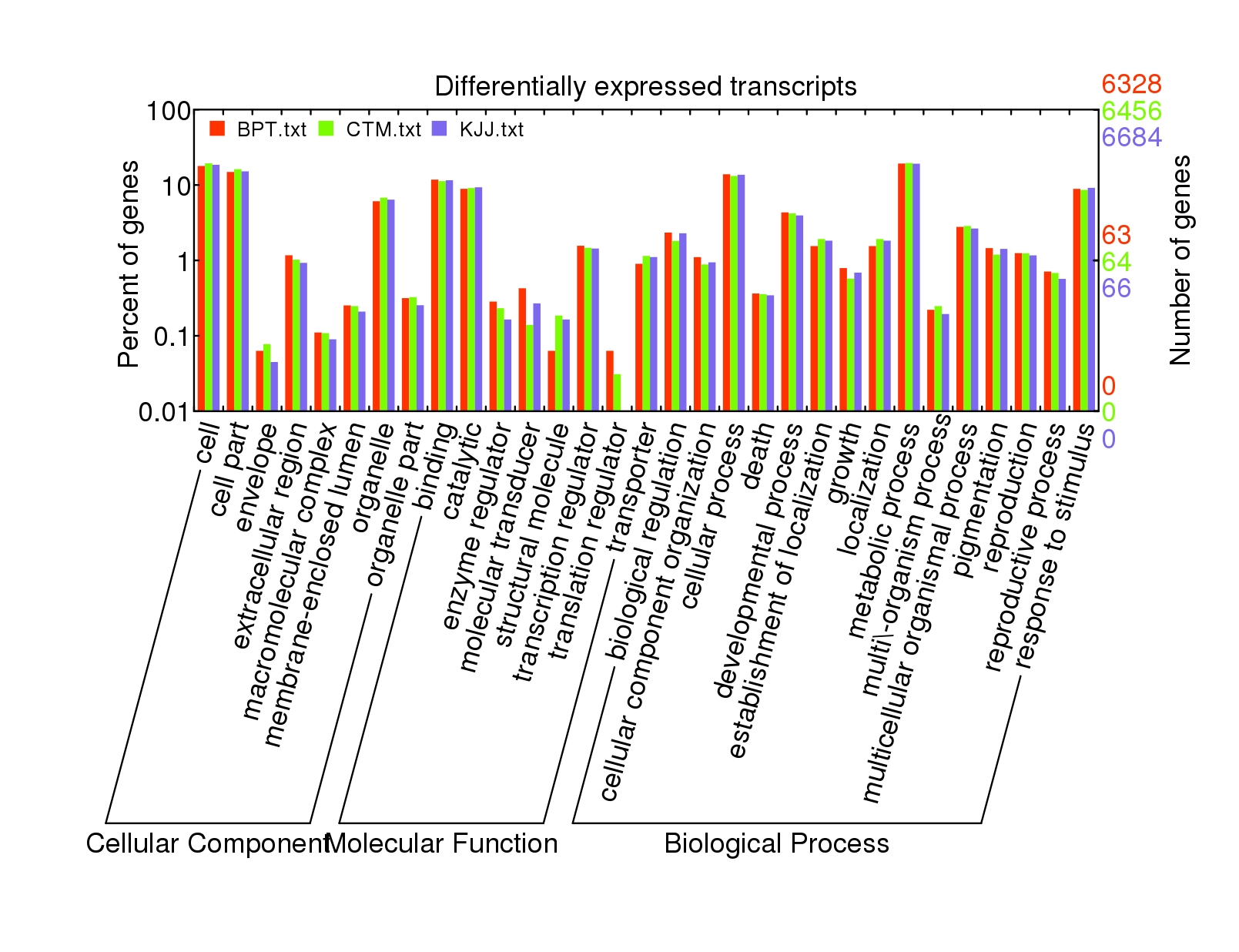

Supplement: S3 Fig — (TIF) [file pone.0192362.s003.tif]
